# Variability in the distance between the suprascapular notch with the spine of the scapulae and the acromion

**DOI:** 10.1007/s10067-023-06807-1

**Published:** 2023-11-08

**Authors:** Jesse Filkin, Nicola Massy-Westropp, Harsha Wechalekar

**Affiliations:** 1 Undergraduate Bachelor of Medical Radiation Science (Nuclear Medicine), University of South Australia, City East Campus, Adelaide, Australia; 2 UniSA Allied Health and Human Performance, University of South Australia, City East Campus, Level 8 Centenary Building, North Terrace, 5000 Australia; 3 UniSA: Allied Health and Human Performance, University of South Australia, North Terrace 5000, City East Campus, Adelaide, Australia

**Keywords:** Acromion, Spine of scapula, Suprascapular nerve block, Suprascapular notch

## Abstract

**Introduction:**

The suprascapular notch lies in the superior border of the scapula and is a passageway for the suprascapular nerve that is sensory to the shoulder joint. Suprascapular nerve block involves injection of local anaesthetic into the suprascapular notch, either ultrasound guided or blind, using the spine of scapula and/or the medial border of the acromion as surface landmarks.

**Aim:**

To investigate the anatomic variations that exist between the distance of the notch from the spine of scapula and acromion.

**Method:**

Ninety-two dry scapulae were measured with a digital calliper for their length of the spine, distance between the midpoint of the spine and base of the suprascapular notch and distance between the medial border of the acromion and the base of the suprascapular notch. These measurements were compared for variations in the scapular bony landmarks, the spine and the acromion to determine the site for the injection.

**Results:**

Measurement reliability was assessed by intraclass correlation, Cronbach’s alpha being 0.99, 0.97 and 0.91 for length of spine, distance from spine and distance from acromion respectively. The distance from the acromion had less variation in measurement (3.73 ± 0.42 cm) but a flatter distribution when compared to distance from the spine of the scapula (3.32 ± 0.39 cm).

**Conclusion:**

Length of the spine of the scapula appeared not to influence either distance from the acromion or distance from the spine of scapula. There is potential for greater variability in placement of nerve blocks that use acromion as the bony reference.

**Table:** 

**Key Points** • *Dry scapular measurement using electronic Vernier callipers is accurate (0.91–0.97).* • *There is potential for greater variability in placement of blind nerve blocks that use acromion as the bony reference to locate the suprascapular notch.*

## Introduction

Shoulder pain is a highly prevalent affliction that accounts for 1.2% of visits to general practitioners [1, 7]. The shoulder is a complex joint with extensive range of movement that attributes to its frequent involvement in dislocations, tears and fractures [3]. The shoulder is also affected in inflammatory conditions like arthritis, bursitis and tendinitis [4]. Shoulder pains, in many cases, are self-limiting; however, in some instances, the pain is so severe that the patients become disabled. The preferred modality of treatment for such irretractable shoulder pain is the suprascapular nerve block (SSNB).

The suprascapular nerve (SSN) arises from the superior trunk of the brachial plexus, C5 and 6, and occasionally from C4 nerve roots, passing through the suprascapular notch to provide motor innervation to the supraspinatus and infraspinatus muscles and sensory innervation to 70% of the shoulder joint capsule [6]. SSNBs involve injection of local anaesthetic into the suprascapular notch to block the SSN for the purpose of reducing shoulder joint pain [5]. Since the invention of the technique, SSNBs are used for treating acute and chronic shoulder pains.

There are different approaches for SSNBs, the surface landmark and imaging techniques. In the surface landmark technique, SSN is accessed by a posterior or a superior approach. In the posterior approach [12, 16], the SSN is blocked in the suprascapular notch while in the superior approach [9], the drug is infiltered in the supraspinous fossa. In either approaches, scapular bony landmarks are used to access the SSN. The posterior approach technique was first described by Wertheim and Rovenstine in 1941 [27]. They used three reference lines, the first line parallel to the upper edge of the spine of scapula extending from the tip of the acromion to the root of the spine, the second from the inferior angle of the scapula bisecting the first and finally the third line bisecting the upper and outer triangle thus formed. The site of injection was chosen on the third line 1.5 cm from the angle. Since then, the posterior surface landmark techniques have undergone several modifications specially to reduce the risk of pneumothorax and vascular complications. This was achieved by adjusting the angulation of the needling. In 2008, Checcucci technique was introduced wherein a point 2 cm medial to the medial border of the acromion and 2 cm cranial to the superior margin of the spine of the scapulae were used for the injection. The needle was introduced until the contractions of the supraspinatus and infraspinatus muscles were elicited [8]. In 2009, Matsumoto et al. used midpoint of the anterolateral angle of the acromion and the medial edge of the scapular spine as the bony landmarks. The needle was directed dorsally with an inclination of 30° from the body axis until it reached the base of the coracoid process [15]. Furthermore, repositioning of the patient position by moving the affected shoulder to the opposite shoulder was also suggested to reduce the postsurgical complications [17]. Superior approach was introduced in patients who were unable to sit for the procedure [9] by introducing the needle parallel to the scapular blade into the supraspinous fossa, a technique that was suggested to be easy and safe without the need to locate the suprascapular notch.

The accuracy of the anterior and superior blind approaches was improved by incorporating imaging techniques like fluoroscopy[22], ultrasound [13–15] or CT scan [20]. Amongst the imaging techniques, ultrasound-guided approach is the most popular because of minimal radiation exposure. A comparison of the surface landmark technique and ultrasound-guided approach showed that both the techniques are comparable and effective in reducing acute and chronic shoulder pains [14]. Scapular bony landmarks are not only a requirement for blind techniques but are also important for positioning of the transducer for ultrasound. While ultrasound visualisation reduces the risk of lung or vascular puncture, not all practitioners have access to the technology.

SSNB is generally a low-risk procedure; however, patients are still at risk of vascular puncture or pneumothorax [5, 16, 17] particularly with the suprascapular vessels lying directly superior to the nerve and the apical part of the lungs anteriorly. As these two complications are both heavily dependent on the location of the injection, it is important that any medical expert performing the procedure places the injection accurately. Location of the suprascapular nerve within the suprascapular notch can vary in individuals because of the variations in the bony scapular landmark that are used for SSNB like the spine of the scapula, acromion and the coracoid process. Numerous studies have investigated the variation in size and shape of the suprascapular notch [3] and have also described six unique types of notches [19]; however, no study investigates the variation in commonly used bony scapular landmarks like the length of the spine of scapula or distance between the coracoid process and the acromion from the suprascapular notch that is necessary for SSNB. Hence, to better understand and quantify the variation in the scapular bony landmarks between individuals, we sought to investigate the variability in scapular measurements that are commonly used when performing SSNBs.

## Method

A quantitative, observational methodology was adopted.

### Scapular measurements

Dry bone scapulae were sourced from University of South Australia and Flinders University. This study was approved by the respective Ethics Committees of the University of South Australia and Flinders University under the body donation program for research using human biospecimens. Dry bone scapulae were measured using an electronic Vernier calliper with a precision of three decimal points (cm). Three scapular measurements were taken to estimate the variation, if any, between the two nerve block approaches.

**Fig. 1:**
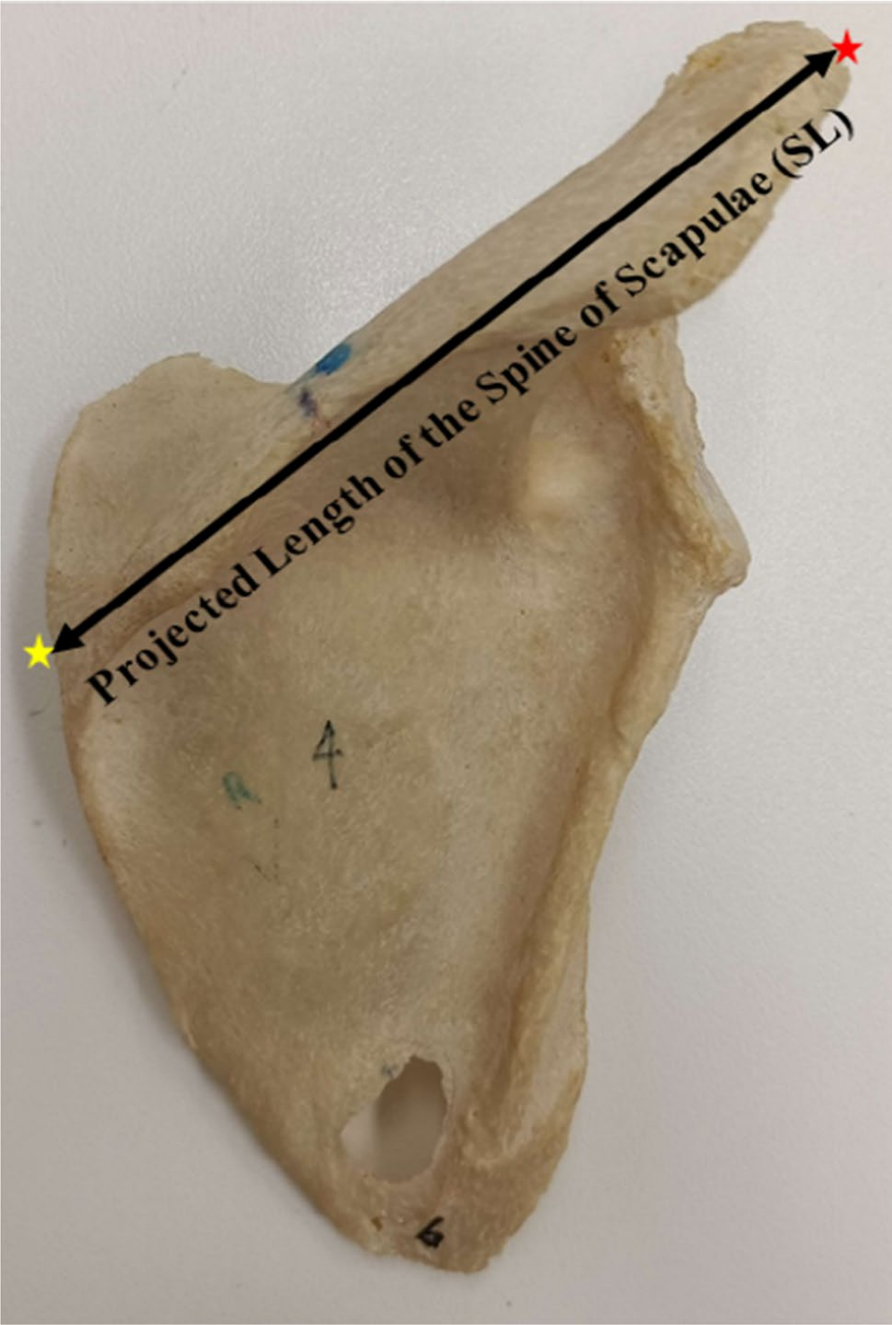
Projected length of the spine of scapulae (SL represented by a black line with arrow tips). SL is the distance from the midpoint of the root of the spine of the scapula (yellow asterisk) to the apex of the curvature of the acromion process at the lateral border (red asterisk)

**Fig. 2:**
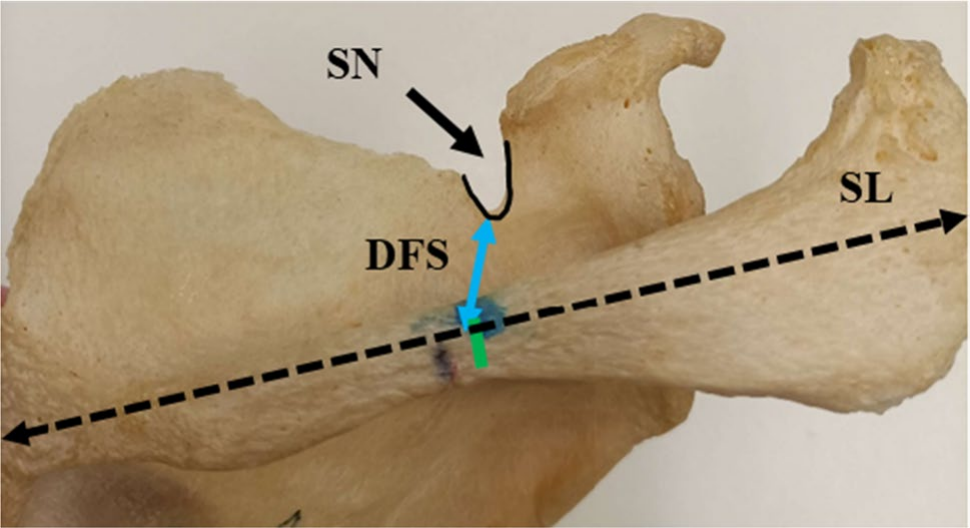
Shows the DFS measurement from the midpoint (green line) of the projected length of the spine of the scapula (SL- dotted line) to the base of the suprascapular notch (SN)

**Fig. 3:**
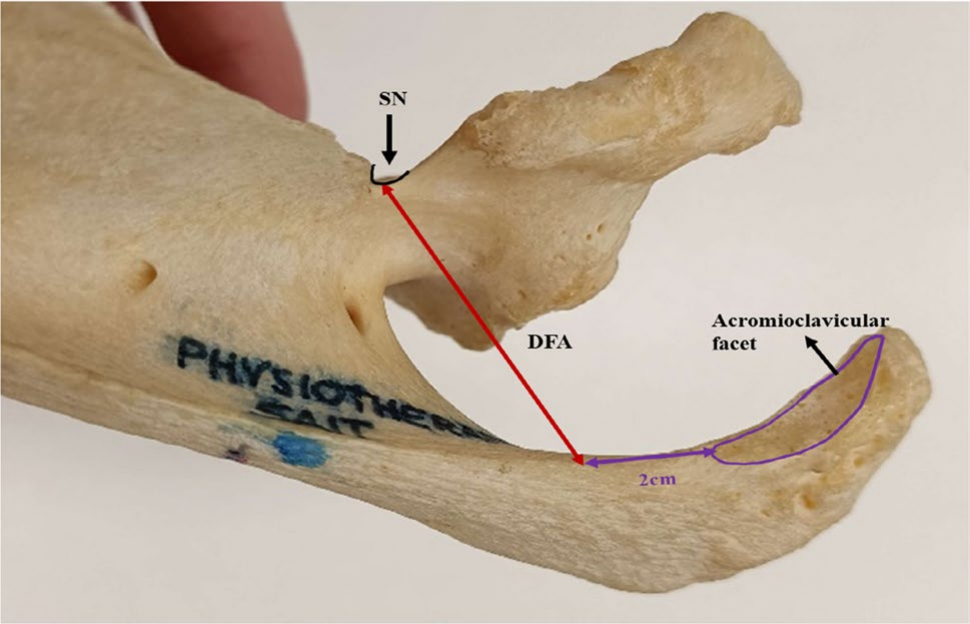
Shows the DFA measurement (red double arrowed line) from a point 2 cm from the medial tip of the acromioclavicular facet (encircled in violet) to the base of the SN

Length of the projected spine of scapulae (SL) was measured from the midpoint of the root of the spine to the apex of the curvature of the lateral border of the acromion process (Fig. 1). Distance from the spine (DFS) was measured from the midpoint of the SL to the base of the SN (Fig. 2), and the distance from the acromion (DFA) was measured from a point 2 cm from the medial tip of the acromioclavicular facet to the base of the SN (Fig. 3).

### Statistical analyses

A power calculation from 10 scapulae was performed to determine the sample size. To achieve 80% power, a sample size of 90 scapulae gave a confidence interval (at *p* < 0.05) width of 0.124 and 0.223 cm for the DFS and DFA measurements respectively. Hence, a total of 92 scapulae were adequate for accurately calculating the variability in the scapular measurements.

To determine the variability, if any, resulting from human error, intrarater reliability was calculated using the Statistical Package for the Social Sciences (SPSS) software to find intraclass correlation (ICC). For ICC, 15 measures were made using five scapulae measured twice each, and intrarater reliability was obtained by Cronbach’s alpha for all the measurements.

Further analysis was done by estimating the mean for SL, DFS and DFA, and the standard deviations of each were utilised to construct and represent the distribution of the data. *T* tests were used to compare the means and distributions of both DFS and DFA. Levene’s test for equality of variances was used to determine whether there was a statistically significant difference in variation of measurements between DFS and DFA. SPSS was used to obtain a correlation coefficient for SL compared to both DFS and DFA, to determine whether SL influences either value. All calculations and analysis were performed with a 95% confidence interval.

## Results

**Fig. 4:**
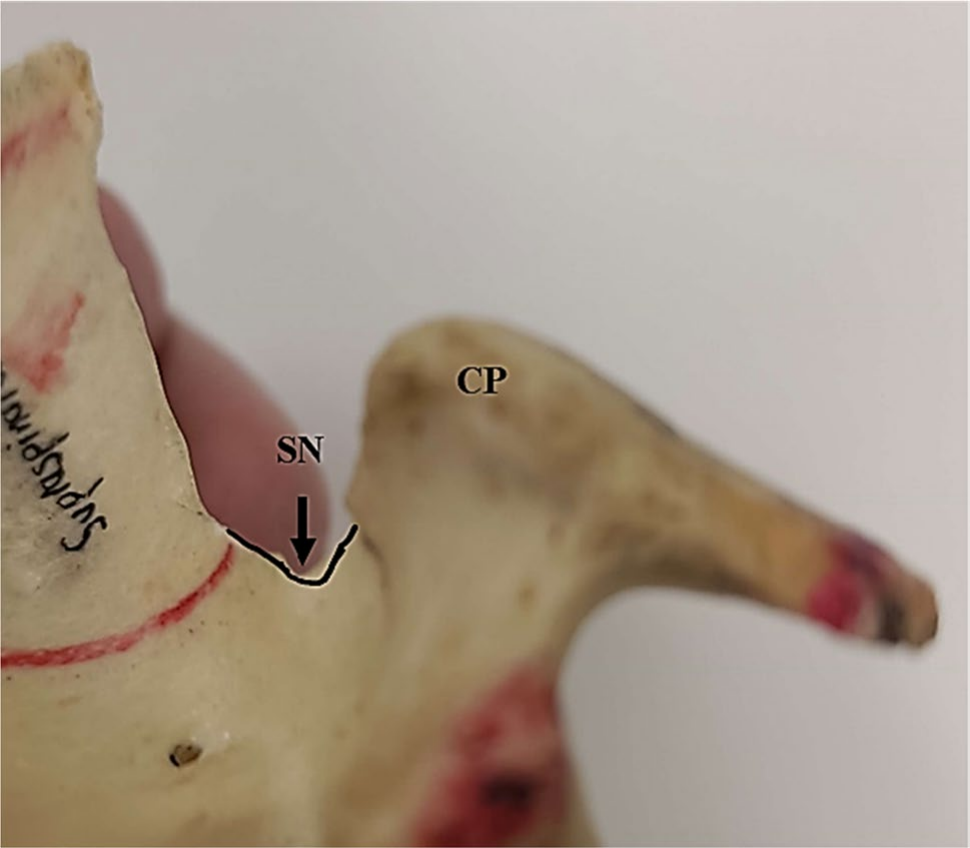
Anterior view of one of the scapulae with morphologically different suprascapular notch. The suprascapular notch (SN) is ‘V’ shaped. CP coracoid process

Of the 92 specimens, there were 44 right and 48 left scapulae. Most scapulae measured were isolated scapular bones; however, 15 pairs measured were from articulated skeleton. There were two scapulae with morphologically different suprascapular notches. These scapulae had notches that were ‘V’ shaped and blunt (Fig. 4).

Measurements of the lengths of SL, DFS and DFA of 92 scapulae showed that the distance of the base of the suprascapular notch from the acromion was greater (3.73 ± 0.42 cm) than its distance from the midpoint of the spine of the scapulae (3.32 ± 0.39 cm) (Table 1). The reliability of all the measurements was assessed by intrarater intraclass correlation coefficient (ICC) by Cronbach’s alpha measures. The Cronbach’s alpha measures were 0.99, 0.97 and 0.91 for SL, DFS and DFA measurements respectively.

**Table 1:** Descriptive statistics of all the scapular measurements and the comparisons of the means of the measurements

Scapular measurements (*n* = 92)	Ranges in length (cm)	Mean (SD)
Projected length of the spine (SL)	10.85–14.83	12.74 ± 0.82
Distance from the spine (DFS)	2.25–4.34	3.32 ± 0.39
Distance from the acromion (DFA)	2.69–4.42	3.73 ± 0.42

The SL and DFS measurements had a normal distribution curve while DFA measurements showed a continuous uniform distribution. Hence, Levene’s *F* test was estimated to look for the equality of the variance between the DFS and DFA measurements [15]. The difference in variance between the two groups was 0.112 (*p* > 0.05). An independent *t* test was performed to look for the differences in the equality of the means between the DFS and DFA measurements. A *p* value of less than 0.001 was estimated between the means of the two measurements (Table 2). In addition, the correlation coefficient was calculated between SL and DFS and SL and DFA measurements. The correlation coefficient between SL and DFS was 0.556 and between SL and DFA was 0.743.

**Table 2:** A summary of the equality of variances between the variables and the means of the measurements. (*f* and *t* are the test statistics, *df* is the degree of freedom and *sig.* is the *p* value corresponding to the test statistics)

DFS and DFA measurements	Levene’s test for equality of variances	*T* test for equality of means
Equal variance assumed	*f* 2.550; sig 0.112	*t* − 6.826
Equal variance not assumed		*t* − 6.826; *df* 181.104 Sig (two tailed) < 0.001

## Discussion

The results show how scapular morphology differs between people thus providing a clinical reference range for the scapular variability for SSNBs. In blind and image-guided SSNBs, distance of the suprascapular notch is measured from the spine of the scapula, acromion and/or coracoid process [11, 18, 23]. However, there are different sizes and shapes of acromion, spine of scapula and the coracoid process. In 1986, Bigliani described three types of acromia in lateral radiographs [6]: type 1 (flat), type 2 (curved) and type 3 (hooked). An increase in slope of the acromion was observed in type 1 to type 3 acromia. Later, a type 4 convex acromion was also described [25]. Studies have also described five types of spines depending on the shape and course of the spine. Type 1, fusiform shape (tapered at both ends and wide in the middle); type 2, slender rod shape (thin throughout); type 3, thick rod shape (thick throughout); type 4, wooden club shape (gradual thickening from medial to lateral edge) and type 5, horizontal S-shape (‘S’-shaped spine) [26]. Similarly, there are five types of coracoid process, type I, vertical 8-shape; type II, long stick shape; type III, short stick shape; type IV, water drop shape, and type V, wedge shape [29]. Whether the shapes and sizes of the acromion, coracoid process and the spine of the scapula affect its distance from the suprascapular notch is not known. It is evident from the literature that for SSNBs, spine of the scapula and the acromion are routinely used bony landmarks for infiltration of the drug into the suprascapular notch. There are only a few studies that have used coracoid process as the reference point [2]. Hence, in the current study, a comparison between two posterior approaches that is distance of the suprascapular notch from the spine and the acromion process was determined. The results showed that the mean value for DFA was higher than DFS. A greater average suggests that DFA is a safer approach for SSNBs as the distance for the needle to traverse the underlying structures is greater thereby decreasing the chance of a complication [21]. Studies have suggested that the acromion process is the most superficial and easiest palpable bony landmark for SSNBs [10]. Although the differences between the two posterior approaches appears to be small but a procedure demanding high precision, any minute difference is also relevant.

In the current study, DFS displayed a lower minimum length of 2.256 compared to DFA measurements with the lowest value of 2.697, and the lower maximum of 4.345 versus 4.428. DFA had a greater mean and standard deviation, while DFS displayed a larger range, with a difference of 2.089 cm between the highest and lowest values, compared to 1.749 cm for DFA. Despite DFS having a slightly greater range, it was more consistent as evident from the normal distribution graph. DFS showed a uniform distribution, which perhaps suggests that DFA is a slightly more variable approach. There was no statistically significant difference in the variance between DFS and DFA approaches, despite differences in the distribution patterns. Our findings show a statistically significant difference in the means of the DFS and DFA (*p* < 0.001). A higher mean value of DFA suggests that measurements from the acromion process are more consistent for blinded approach in SSNBs.

To determine the degree to which measurements may have fluctuated because of user error, intraclass correlation was determined by Cronbach’s alpha measures. A high correlation between the original measurements and the repeated measurements was observed. Generally, on a scale of zero to one, a value of one represents maximum internal consistency [24]. In the current study, Cronbach’s alpha was greater than 0.9 for all three measurements, indicating high reliability.

The current study showed a moderate correlation between SL and DFS with correlation coefficient of 0.556 and a greater correlation coefficient of 0.743 between SL and DFA measurements. As DFS is taken from the midpoint of the spine of scapulae, SL length would be expected to have a lesser influence when compared to DFA which is taken from the medial end of the acromioclavicular facet on the acromion. Additionally, DFA’s greater influence from SL may be associated with the varying curvatures of the acromion where it meets the spine [4],with some scapulae bending away from the notch at that point. These findings indicate that patients with larger scapular spines would likely benefit more from the DFS approach as it is less susceptible to variation from spine length. Two of the scapulae in the cohort had variant suprascapular notches. These V-shaped notches blended with the superior border of the scapula, resulting in slightly greater DFS and DFA than other scapulae. These suprascapular notches [19] pose a high risk of vascular punctures in SSNB blocks; however, they are less common than other notch types.

In the current study, the gender of the scapulae was not included; however, there is a likelihood of impact of gender on DFS and DFA measurements. Studies have shown that it is possible to determine male and female scapulae [28]. Scapulae from male are larger with a larger glenoid cavity that can directly influence SL, DFS and DFA within our investigation. Determination of gender was omitted as a variable in the study due to time constraints of the project.

Studies have shown that SSNB is an effective modality of treatment for reducing shoulder pain [14]; however, further research may help to avoid complications such as vascular trauma and pneumothorax. While differences have been discovered in the two SSNB techniques, one approach has not been clinically proven safer than the other.

Regardless of the size of impact scapular variations may have on blind SSNB, and the differences between DFS and DFA, these results may provide a reference for clinicians and support future investigations regarding SSNB.

## Data Availability

Deidentified data will be considered to be provided upon receipt of a pertinent varification/research request.
